# Supplementary material for: Cellular-resolution OCT reveals layer-specific retinal mosaics and ganglion cell degeneration in mouse retina in vivo
Source: Neurophotonics. 2026 Jan 23;13(1):015006. doi: 10.1117/1.NPh.13.1.015006 (PMC12830085; doi:10.1117/1.NPh.13.1.015006)
Supplement: Supplementary file 1 [file NPh_013_015006_SD001.pdf]

Supplementary Information for

Cellular-resolution OCT reveals layer-specific retinal mosaics and ganglion cell degeneration in mouse retina in vivo

Tae-Hoon Kim[1], Robby Weimer[1], Justin Elstrott[1,*]
[1]Department of Translational Imaging, Genentech, Inc., CA 94080, USA

Correspondence: *Justin Elstrott, Department of Translational Imaging, Genentech, Inc., 1 DNA Way, South San Francisco, CA 94080, USA
Tel: +1-650-225-8580, Email: elstrott.justin@gene.com

This PDF file includes:
Supplementary Figures S1 to S3.
Legends for Movie S1.

Other supplementary materials for this manuscript include the following:
Movie S1.mpeg

Input: A set of N initial OCT volume files
Output: A single, final aligned and averaged volume

Let $V_{current}$ be the initial set of N volume files
while number of volumes in $V_{current} > 1$ **do**
    Let V_{next_level} be an empty list
    for each batch of volumes in $V_{current}$ **do**
        Load and preprocess volumes
Stage 1: Rigid Registration (XZ Plane)
        Compute side-projection from central 60 B-scans
        Perform rigid registration of side-projections
        Apply transformations to original volumes
Stage 2: En Face Registration (XY Plane)
        Generate En Face projections and apply translational alignment
Stage 3: B-scan Alignment (Axial)
        Concatenate volumes into an interleaved volume
        Apply vertical displacement correction to B-scans
Averaging and Saving for the Batch
        Average aligned frames to produce $V_{averaged}$
        Save the averaged volume $V_{averaged}$
        Add $V_{averaged}$ to list V_{next_level}
    end for
    $V_{current} \leftarrow V_{next\_level}$ ▷ Set outputs as inputs for next level
end while
return the single volume remaining in $V_{current}$

Supplementary Figure S1. Hierarchical multi-volume registration and averaging algorithm. The iterative pipeline processes OCT volumes through three-stage registration (rigid XZ-plane, en face XY-plane, and axial B-scan alignment) followed by averaging to progressively reduce volume count while enhancing image quality through speckle noise suppression.

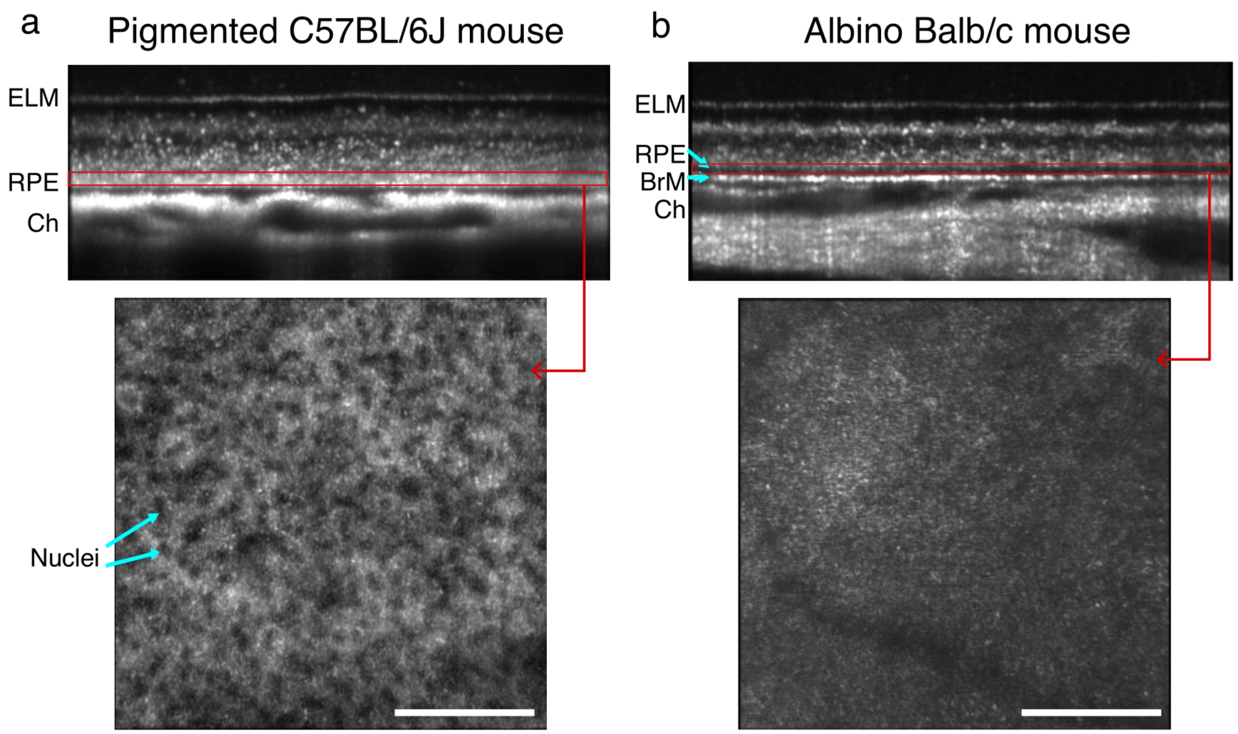

Supplementary Figure S2. The RPE mosaic pattern is visible only in pigmented mice. Representative cross-sectional (top) and *en face* (bottom) OCT images of the RPE layer in (a) a pigmented C57BL/6J mouse and (b) an albino Balb/c mouse. In the pigmented mouse, the *en face* image reveals a distinct mosaic pattern of regularly spaced, hyporeflective spots, presumed to be nuclei surrounded by hyperreflective melanosomes. This cellular mosaic is absent in the non-pigmented RPE of the albino mouse. (ELM, external limiting membrane; RPE, retinal pigment epithelium; BrM, Bruch's membrane; Ch, Choroid). Scale bars: 100 µm.

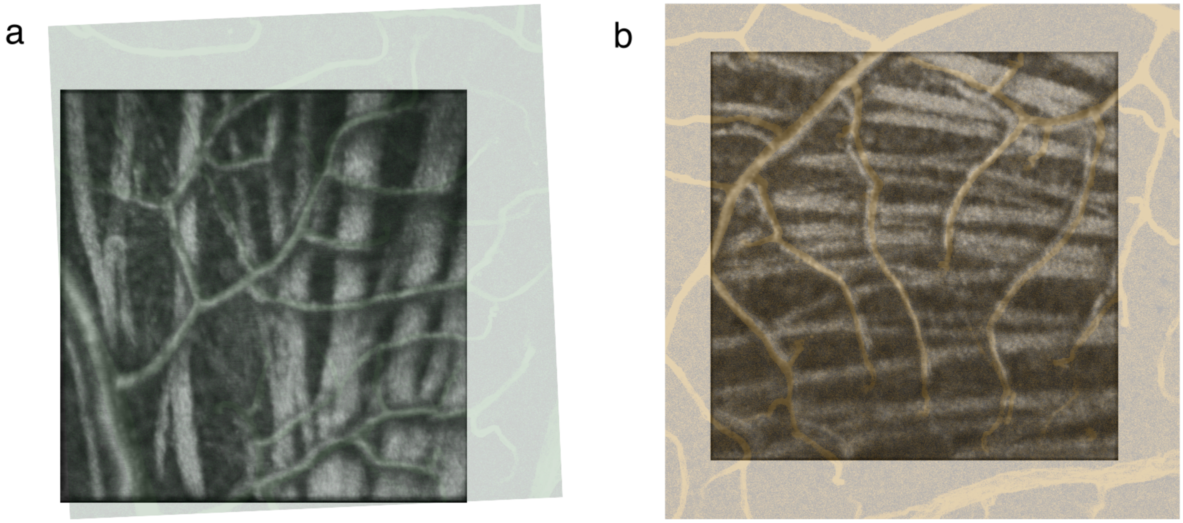

Supplementary Figure S3. Calibration of the OCT field of view (FOV) using co-registered confocal images of retinal vasculature. To determine the precise FOV of the in vivo OCT imaging, a whole-mount retina was stained for vasculature with a CD31 marker and imaged via confocal microscopy. The resulting confocal image, which has a known physical size of 388 × 388 µm, was then overlaid onto the corresponding OCT image using the vascular pattern for alignment. The overlay is shown for two independent samples using different pseudocolors for visualization purposes: green in (a) and yellow in (b). By using the known dimensions of the confocal image as a calibrated ruler, the FOV of the underlying OCT image was estimated to be approximately 310 × 310 µm, which corresponds to roughly 8 x 8 degrees of the adult mouse visual field.

Supplementary Movie S1. In vivo visualization of cellular mosaics in four distinct retinal layers. This supplementary file consists of four separate movies, each presenting an axial fly-through within a specific retinal layer of a living mouse. The movies display the distinct cellular mosaics of the retinal ganglion cell (RGC) layer, the inner nuclear layer (INL), the outer nuclear layer (ONL), and the retinal pigment epithelium (RPE), respectively. Together, these visualizations demonstrate the capability of the integrated workflow to resolve individual cells with their unique layer-specific morphologies in vivo. Scale bar, 100 μm.
